# Differentiation of Parasitized and Non-parasitized *Anastrepha fraterculus* (Wiedemann, 1830) (Diptera: Tephritidae) Pupae Based on Hyperspectral Reflectance Profiles

**DOI:** 10.1007/s13744-026-01423-5

**Published:** 2026-08-19

**Authors:** Eduarda Bender, Simone Mundstock Jahnke, André Luís Vian, Daniel Capella Zanotta

**Affiliations:** 1 Posgraduate Program in Plant Science, Faculty of Agronomy, Federal University of Rio Grande Do Sul (UFRGS), Porto Alegre, Rio Grande Do Sul, Brazil; 2 Center of Excellence in Geoinformatics and Visual Computing, University of Vale Do Rio Dos Sinos, São Leopoldo, Rio Grande Do Sul, Brazil

**Keywords:** Biological control, parasitism, *Diachasmimorpha longicaudata*, random forest model, development times (DD)

## Abstract

Biological control programs for fruit flies rely on the mass rearing and release of parasitoids to suppress pest populations. The success of this strategy depends on effective quality control, including the assessment of parasitism and insect survival. Conventional methods, such as dissection, Polymerase Chain Reaction (PCR), and adult emergence, are destructive, labor-intensive, and time-consuming. Hyperspectral imaging offers a rapid and non-destructive alternative for evaluating parasitism. Therefore, this study aimed to discriminate *Anastrepha fraterculus* (Wiedemann, 1830) (Diptera: Tephritidae) pupae parasitized by *Diachasmimorpha longicaudata* (Ashmead, 1905) (Hymenoptera: Braconidae) using hyperspectral imaging and identifying the most informative spectral ranges and wavelengths for this purpose. Hyperspectral images were acquired from pupae at different developmental stages expressed in degree days (DD), preserving insect viability. Principal component analysis (PCA) and clustering analysis indicated a tendency to separate parasitized from non-parasitized pupae. Spectral profiles showed lower reflectance in the visible region and higher reflectance in the near-infrared (NIR, > 700 nm) for parasitized pupae. Greater spectral variability was also observed throughout development, particularly at 7 and 12 days (135 and 189 DD, respectively), compared with non-parasitized pupae at the same chronological ages (260.1 and 321.3 DD, respectively), which exhibited a more homogeneous spectral pattern. The Random Forest model showed moderate classification performance across all developmental stages, with higher predictive accuracy at later stages. These findings demonstrate the potential of hyperspectral imaging as a non-destructive tool for detecting parasitism and improving quality control in mass-rearing programs for biological control.

## Introduction

The biological control programs for fruit flies are based on the mass production and release of parasitoids in the field, aiming to significantly impact the population and reduce it to economically acceptable levels (Freire et al. 2005; Van Lenteren et al. 2021). For these programs to be successful, rigorous quality control in mass rearing is essential. This involves assessing various parameters, including host preference and suitability, flight capability, parasitoid searching behavior, parasitism efficiency, host adaptation, mating competitiveness, and overall survival (Prezotti and Parra 2002).

Assessing the quality of fruit flies and parasitoid pupae produced through mass rearing offers key advantages, as it allows for accurate determination of parasitism rates and helps minimize the risk of releasing fruit flies in the field (Cancino and Montoya 2006; Mastrangelo et al. 2019; Marinho et al*.*^2023^). It is important to note that parasitism rates in mass rearing rarely reach 100% due to several factors (Marinho et al. 2023). The percentage of pupae and the parasitism rate in each lot can only be determined through the dissection of the pupae, Polymerase Chain Reaction (PCR) to detect parasitoid DNA sequences, or by waiting for the parasitoids to emerge (Cancino and Montoya 2006; Gariepy et al. 2007).

The mechanical separation of pupae in each lot is a labor-intensive and time-consuming process that often causes damage to the insects (Marinho et al. 2023). The irradiation of larvae or pupae before field release has been adopted to reduce the risk of releasing fruit flies (Ramadan et al. 1989; Camargos et al. 2018). As an alternative, imaging spectroscopy could become an effective tool in this context (Zahra et al. 2023). These include multi and hyperspectral sensors that cover more than the three spectral bands typically used in visible spectrum imaging (Zahra et al. 2023).

Spectral imaging has been reported for identifying the presence of parasitoid *Spalangia cameroni* (Perkins, 1910) (Hymenoptera: Spalangiidae) or *Muscidifurax raptorellus* (Girault and Sanders, 1910) (Hymenoptera: Pteromalidae) in the puparium of *Musca domestica* (Linnaeus, 1758) (Diptera: Muscidae) using NIR (700 to 1700 nm) (Dowell et al. 2000). Also, three parasitoid species from the genus *Trichogramma* (Westwood, 1833) (Hymenoptera: Trichogrammatidae) were successfully identified based on spectral data (405–907 nm) obtained from *Ephestia kuehniella* (Zeller, 1879) (Lepidoptera: Pyralidae) eggs parasitized by these species (Nansen et al. 2014).

Originally developed for satellite monitoring and facial recognition, spectral imaging technologies have also shown remarkable effectiveness in identifying agricultural pests and diseases (Nansen and Eliot 2016; Zanotta et al. 2019). These tools allow for rapid, non-invasive assessments of food quality and safety (Choi et al. 2021) and have been used, for example, to evaluate grain quality and differentiate fresh rice grains from those damaged by insect pests such as weevils (Srivastava and Mishra 2022). Near-infrared (NIR) spectroscopy has also been employed to analyze the cuticle of weevils under varying nutritional conditions (Lacotte et al*.*^2023^). In addition, NIR spectroscopy enables accurate, non-destructive sexing of *Glossina pallidipes* (Austen, 1903) (Diptera: Glossinidae) pupae, with optimal discrimination occurring 4–5 days before adult emergence. This early identification, effective within the 950–1700 nm spectral range, provides sufficient time for sterilization and logistical preparation in sterile insect technique (SIT) programs (Dowell et al. 2005).

Considering the potential of hyperspectral imaging, our study aimed to a) determine the optimal development time of pupae in degree days (DD) for acquiring discriminative images; b) establish the reflectance patterns of non-parasitized and *Diachasmimorpha longicaudata* (Ashmead, 1905) (Hymenoptera: Braconidae) parasitized fruit fly pupae; c) identify the most suitable bands and ranges for differentiating between the pupal states.

## Material and methods

### Insect rearing

Insect rearing was maintained at the Insect Biological Control Laboratory (CBLAB), Faculty of Agronomy at the Federal University of Rio Grande do Sul (UFRGS). The insects were kept under controlled conditions at a temperature of 25°C ± 1°C, relative humidity (RH) of 60% ± 10%, and a photoperiod of 14:10 h (L:D).

#### *Anastrepha fraterculus* flies

Adults of *A*. *fraterculus* sensu lato were kept in cages with a wooden frame (45 × 30 × 30 cm) and sides covered with voile fabric, featuring a front opening adapted from the same fabric. Females began to lay eggs approximately seven days after emergence and were kept in the cages for up to 30 days (Salles 1995).

The diet provided to the adults was a solid mixture of ad libitum, consisting of Caravelas® sugar, brewer’s yeast, wheat germ, soybean extract in a 3:1:1:1 ratio, and a vitamin complex (Lavitan – AZ®), with two crushed tablets per 250 g of diet (adapted from Jaldo et al. 2001). The mixture was then homogenized and placed in Petri dishes (5 × 5 × 1.5 cm) set in the cages, along with distilled water in 140 mL plastic containers. The water was provided by capillarity through Spontex Resist® fabric strips, which were put into a perforated lid and submerged in the water.

The substrate used for female oviposition consisted of a blue voile bag coated with silicone, one end featured a lidded opening through which water was added. This bag was placed on top of the rearing cage, in contact with the cage's voile fabric (Meirelles et al. 2015). The females oviposited through the cage mesh into the substrate, allowing the eggs to remain submerged in water. A small volume of distilled water (approximately 30 mL) was maintained inside the bag to prevent egg desiccation.

The contents of the bags (eggs and water) were transferred to a 200 mL beaker. Using a pipette, the eggs were collected, spread over a blue voile fabric strip (2 × 6 cm), and subsequently placed on the diet.

The artificial diet for larval development consisted of raw organic carrot (125 g), cooked organic carrot (125 g), brewer’s yeast (25 g), cornmeal (150 g), sugar (125 g), distilled water (175 mL), sodium benzoate (Dinâmica®) (1.1 g), nipagin (Synth®) (1.1 g), and citric acid (Synth®) (3.6 g) (adapted from Terán, 1977). The diet (150 g) was placed in trays (18 × 24 × 2 cm) in which the eggs were deposited on the voile fabric described above.

The trays containing the eggs were covered with an identical tray and wrapped in paper, where the date and rearing details were recorded. They were then stored in Biochemical Oxygen Demand incubator under the same conditions described for rearing, where they remained for seven days. After this period, the trays containing developing larvae were placed inside larger plastic trays (51 × 30 × 9.5 cm); the bottoms of these vessels were covered with a 5 cm layer of sterile sand to serve as a pupation substrate, where they were kept for an additional seven days. Following this period, the pupae were separated from the sterile sand using fine sieves (2.0 mm mesh size). They were then transferred to plastic containers (300 mL) labeled with the corresponding dates. The containers were covered with perforated plastic lids to ensure proper air circulation. The pupae were kept under these conditions until emergence, which occurred at approximately 14 ± 1 day.

### *Diachasmimorpha longicaudata* parasitoid

The adults were kept in wooden cages (19.5 × 16.5 × 25.5 cm) with voile fabric covering the sides and a front opening lined with the same fabric, which allows for the placement of food and the insertion of parasitism units. The diet consisted of honey dissolved in water at a 7:3 ratio, provided in Petri dishes (5 × 5 × 1.5 cm) soaked in cotton, and distilled water offered by capillarity through two strips of Spontex Resist® (Meirelles et al. 2015). Each cage was provided with a parasitism unit containing approximately 50 third instar larvae (about seven days old) of *A. fraterculus*, at a ratio of approximately two larvae per female.

The *A. fraterculus* parasitism unit consisted of a plastic dish (4 cm in diameter) with a 0.3 cm edge, lined with a thin layer of silicone, and covered with white organza fabric secured with an elastic band, as described by Rohr et al. (2019). The units were exposed to parasitoids for 40 to 50 min. Immediately after parasitism, the larvae were placed in plastic trays (41 × 28 × 7 cm) containing a thin layer of sterile vermiculite at the bottom and kept for seven days to allow pupation. The resulting pupae were stored in plastic containers (140 mL) and maintained in climate-controlled chambers until adult emergence.

### Obtaining the Pupae

The parasitized puparia of *A. fraterculus* were obtained as described in the previous item. The puparia were initially inspected for the presence of puncture marks prior to image acquisition. After the imaging procedure, the puparia were maintained under controlled environmental conditions until adult emergence. Emergence and sex of both host flies and parasitoids were subsequently confirmed. For the analysis, only puparia that successfully yielded adult parasitoids were considered and classified as parasitized. Puparia from which host adults emerged were excluded from the parasitized group, while those that showed no adult emergence, including dead or contaminated specimens, were excluded from the analysis.

To obtain non-parasitized *A. fraterculus* puparia, third instar larvae were collected from the artificial diet, transferred to parasitism units, and placed inside rearing cages under the same environmental conditions and during the same period as the parasitized group, but without exposure to parasitoids. The resulting puparia measured approximately 4.5–6.0 mm in length and 2.0–2.5 mm in diameter. They were cylindrical, with a yellowish-red to dark brown coloration.

The pupae were stored in containers with sterile sand, and the day of pupation was recorded to calculate developmental time in degree-days (DD). All insects were reared under the same environmental conditions, with controlled temperature and relative humidity. Consequently, the puparia were evaluated at the same chronological time (in days), although they were not necessarily at the same developmental stage in terms of DD, as the organisms studied exhibit distinct thermal requirements. Therefore, degree-days (DD) were calculated individually for each organism to describe their respective thermal developmental trajectories, rather than as a criterion for physiological equivalence between parasitized and non-parasitized pupae.

### Degree-days (DD) of pupae

The accumulated degree-days (DD) for the egg-to-adult period for *A. fraterculus* were determined based on the daily mean air temperature by summing the daily thermal units above the base temperature, as described by Haddad et al. (1999):

DD=y.(T-Tb)

where, DD: accumulated thermal units per day; y: time required to complete development (days); T: mean daily air temperature; Tb: base temperature for the egg-to-adult period of the fly and the parasitoid *D. longicaudata.*

Based on the development time at a mean temperature of 26°C, non-parasitized *A. fraterculus* pupae were evaluated from the egg to the pupal stage at approximately 260.1, 290.7, and 321.3 DD. In contrast, parasitized pupae were evaluated at 135.0, 162.0, and 189.0 DD.

### Image acquisition

Groups of five non-parasitized and parasitized *A. fraterculus* puparia were placed separately on a pink cardstock piece (3 × 1 cm) and positioned in the same orientation (dorsal view). For each puparium, a small region of interest (ROI) of approximately 5 × 5 pixels was selected, and the reflectance values were averaged for each spectral band. The ROI was defined as a visually uniform area of the puparia surface, positioned to avoid regions affected by specular reflection. All background pixels were excluded from the analysis.

Although minimal background scattering could remain due to optical effects, the point spread function (PSF) of the sensor is sufficiently narrow to prevent substantial contamination of the measured spectra. This effect is negligible for pixels located within the interior of the puparium, where the ROIs were consistently selected.

Although groups of five puparia were imaged simultaneously, reflectance spectra were extracted individually for each puparium. Regions of interest were defined separately for each individual, ensuring that only pixels corresponding to a single puparium were included in the spectral extraction. Consequently, each puparium was treated as an independent sampling unit within the dataset.

Hyperspectral images were obtained from at least 25 parasitized and 25 non-parasitized puparia of different ages, expressed in DD, as described above. A total of 150 average reflectance profiles were obtained from individual puparium (3 ages × 2 treatments (with/without parasitism) × 25 repetitions. The hyperspectral imaging system consisted of a push-broom hyperspectral camera (Pika L, Resonon Inc®) equipped with a 23 mm objective lens, mounted either 30 or 60 cm above the cardstock holding the puparia.

The camera captured spectral data in the 400–1000 nm range, comprising 281 bands with a spectral resolution of 3.1 nm, a bandwidth of 2.7 nm, and a radiometric resolution of 12 bits. Each line captured 900 spatial pixels. Although the camera has a maximum frame rate of 249 fps, a frame rate of 21 fps was used in this study. Two-dimensional images were created by assembling line-by-line scans as the sample moved relative to the camera on a linear translation stage.

Hyperspectral data were acquired in a dark room maintained at 25 ± 2°C using artificial illumination provided by halogen lamps mounted on a tower. Dark references were obtained by covering the camera lens with a non-reflective black cap, while a white Teflon standard (K-Mac Plastics, MI, USA) served as the white reference. Relative reflectance was determined by comparing the reflectance measured from each sample with the corresponding white and dark references and was expressed relative to the Teflon standard. This correction procedure standardized the spectral data and reduced the influence of minor fluctuations in illumination. Consequently, reflectance values extracted from the hyperspectral image cubes were normalized to a range between 0 and 1. The camera focus was adjusted by loosening the locking metal collar attached to the lens and rotating the focus ring until optimal image sharpness was achieved.

After image acquisition, the puparia were individually placed in 140 mL plastic pots containing a layer (± 1 cm thick) of sterilized sand treated with nipagin (0.01%) and an Eppendorf-type tube filled with distilled water and sealed with a hydrophilic cotton plug to maintain humidity. The pots were labeled according to the image acquisition sequence and stored in a climate-controlled chamber until adult emergence, at which point the sex of the flies and the parasitoids was confirmed.

### Statistical analysis

After processing the images acquired as described above, the spectral data were extracted. For each developmental time (DD), a principal component analysis (PCA) was performed to contrast the overall average reflectance values between the groups (non-parasitized and parasitized). The average spectral variation data for each developmental time (DD) were plotted in relation to different wavelengths (nm) for the VIS and NIR regions. In addition, the average spectral variation at each developmental stage (DD) was plotted across the full spectral range, with reflectance values represented as a function of wavelength (nm).

The classification was performed using the Random Forest machine learning algorithm, a non-parametric supervised method based on ensembles of decision trees, widely employed in spectral analysis and biological datasets due to its intuitive interpretation of results.

The model was applied separately for each developmental time (DD) to capture specific spectral differences associated with distinct puparia ages. The Random Forest was configured with 100 decision trees, a value defined to ensure stability of the estimates and convergence of the error. To guarantee reproducibility, a fixed random seed (42) was used. In the exploratory analysis of variable importance, the 10 most relevant wavelengths were considered, using 15 permutation repetitions.

Model validation was carried out using the out-of-bag (OOB) validation strategy, which is intrinsic to the Random Forest algorithm. During training, each tree was constructed from a bootstrap sample containing approximately 67% of the observations, while the remaining 33% were used as OOB samples for validation. Each observation was evaluated only by trees in which it was not included during training, ensuring adequate separation between training and validation data.

Model performance was estimated based on the OOB error, obtained by comparing predictions made on the OOB samples with their corresponding true labels. The following performance metrics were calculated: accuracy, balanced accuracy, and F1-score.

The contribution of each wavelength to the discrimination between parasitized and non-parasitized puparia was assessed using the internal variable importance metrics provided by the Random Forest algorithm. This analysis was exploratory in nature and aimed to identify spectral regions most informative for classification, while retaining all spectral bands throughout the modeling process.

Model training and data analysis were performed using Python with the scikit-learn library in Python 3 (Phython 2024).

## Results

### Exploratory analysis

**Fig. 1:**
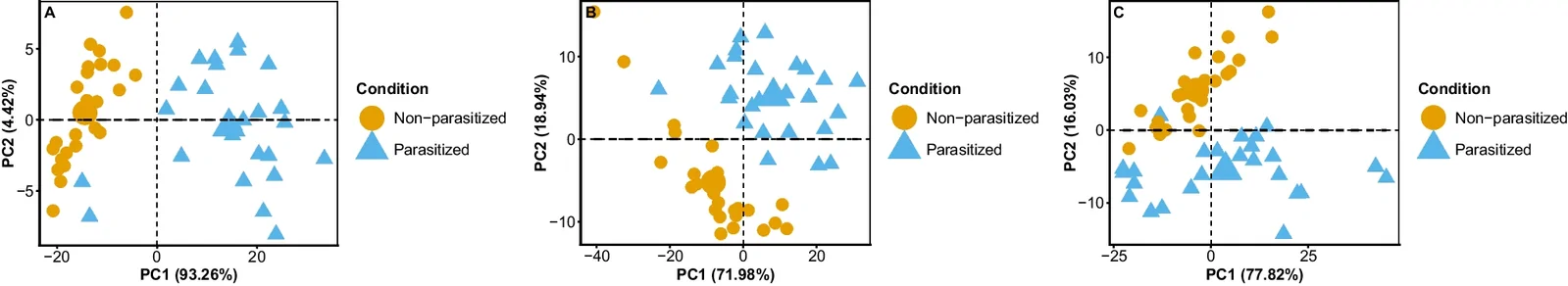
Principal Component Analysis (PCA) of *Anastrepha fraterculus* pupae comparing parasitized by *Diachasmimorpha longicaudata* and non-parasitized individuals at different developmental stages (expressed in degree days, DD): **A** 7 days (135 DD for parasitized, 260.1 DD for non-parasitized), **B** 10 days (162 DD for parasitized, 290.7 DD for non-parasitized), and **C** 12 days (189 DD for parasitized, 321.3 DD for non-parasitized)

The PCA (Fig. 1) was used for exploratory purposes to visualize patterns in the spectral data. The score plots showed an apparent tendency toward separation between pupae parasitized by *D. longicaudata* and non-parasitized pupae, with PC1 accounting for most of the total variance across all evaluated ages (DD). At 7 days (135 DD for parasitized and 260.1 DD for non-parasitized pupae) (Fig. 1A), PC1 explained 93.68% of the variance, reflecting differences in the overall spectral variation and a higher dispersion among parasitized pupae. No formal statistical inference regarding group discrimination was derived from the PCA.

During 10 days of development (162 DD for parasitized and 290.7 DD for non-parasitized pupae) (Fig. 1B), the proportion of variance explained by PC1 decreased to 69.57%, while PC2 accounted for a larger share of the total variance (20.13%). This shift in variance distribution indicates greater multidimensional variability in the spectral data, suggesting that additional pupal attributes contributed to the overall data structure at this stage. At 12 days (189 DD for parasitized and 321.3 DD for non-parasitized pupae) (Fig. 1C), PC1 regained greater influence, explaining 71.68% of the variation, with PC2 accounting for 20.52%, maintaining a well-defined pattern and demonstrating greater variability within the parasitized group.

**Fig. 2:**
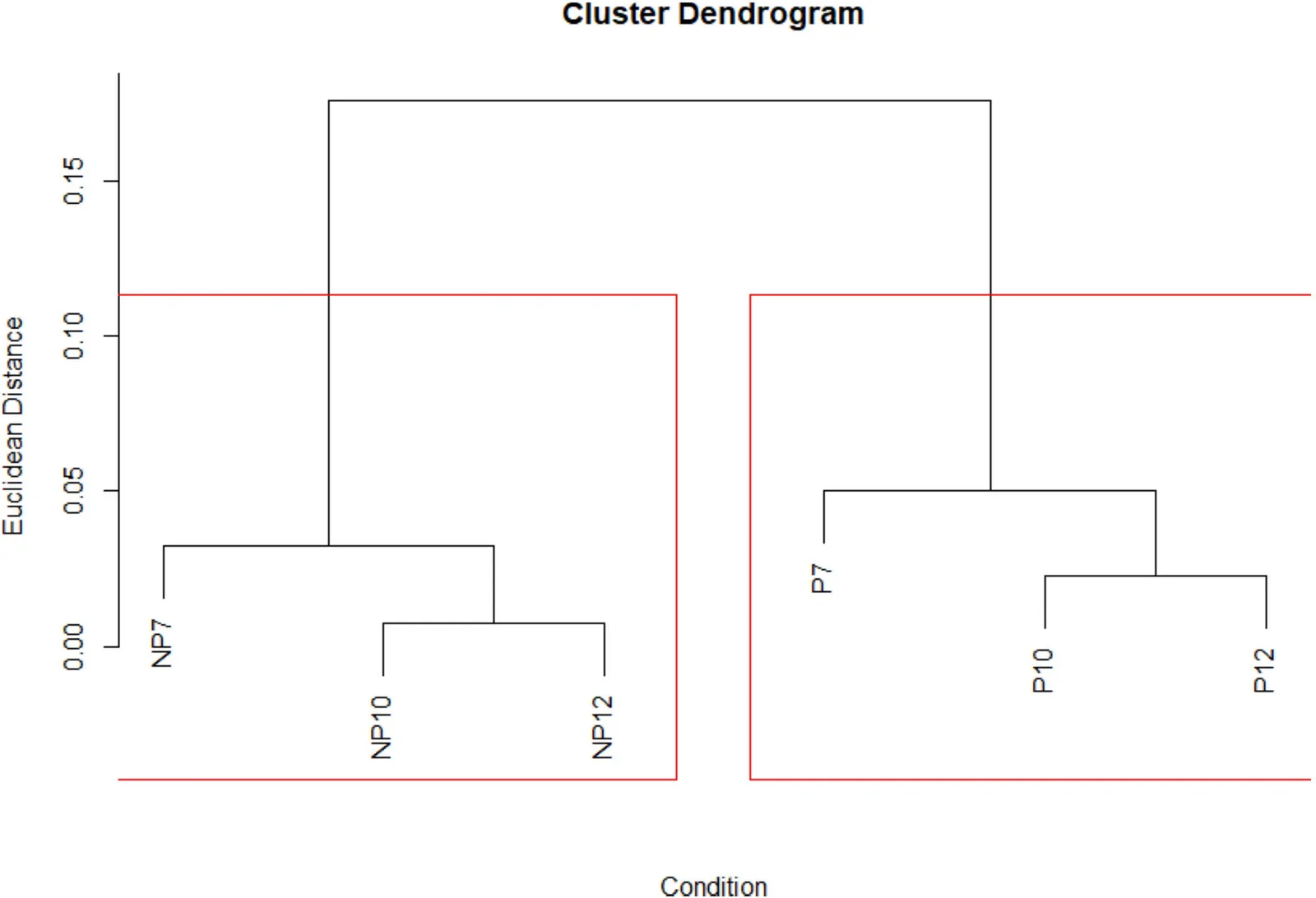
Cluster Dendrogram shows spectral similarity among *Anastrepha fraterculus* pupae. For parasitized pupae (P) by *Diachasmimorpha longicaudata*, 7 days correspond to 135 degree-days (DD); 10 days (162 DD), and 12 days (189 DD). For non- parasitized pupae (NP), 7 days (260.1DD), 10 days (290.7 DD), and 12 days (321.3 DD). *Euclidean distance was used for clustering

Cluster analysis reinforces the patterns observed previously by revealing trends in the organization of the data into two main clusters associated with parasitized and non-parasitized pupae, with a Euclidean distance greater than 0.15 (Fig. 2). The dendrogram also reveals an internal structure related to pupal age within each condition, with 7-day-old pupae showing greater relative dissimilarity in both groups. This pattern suggests that, at this developmental stage, spectral variations are more pronounced, indicating a greater potential for early differentiation between parasitized and non-parasitized pupae.

### Spectral behavior in the VIS/NIR

**Fig. 3:**
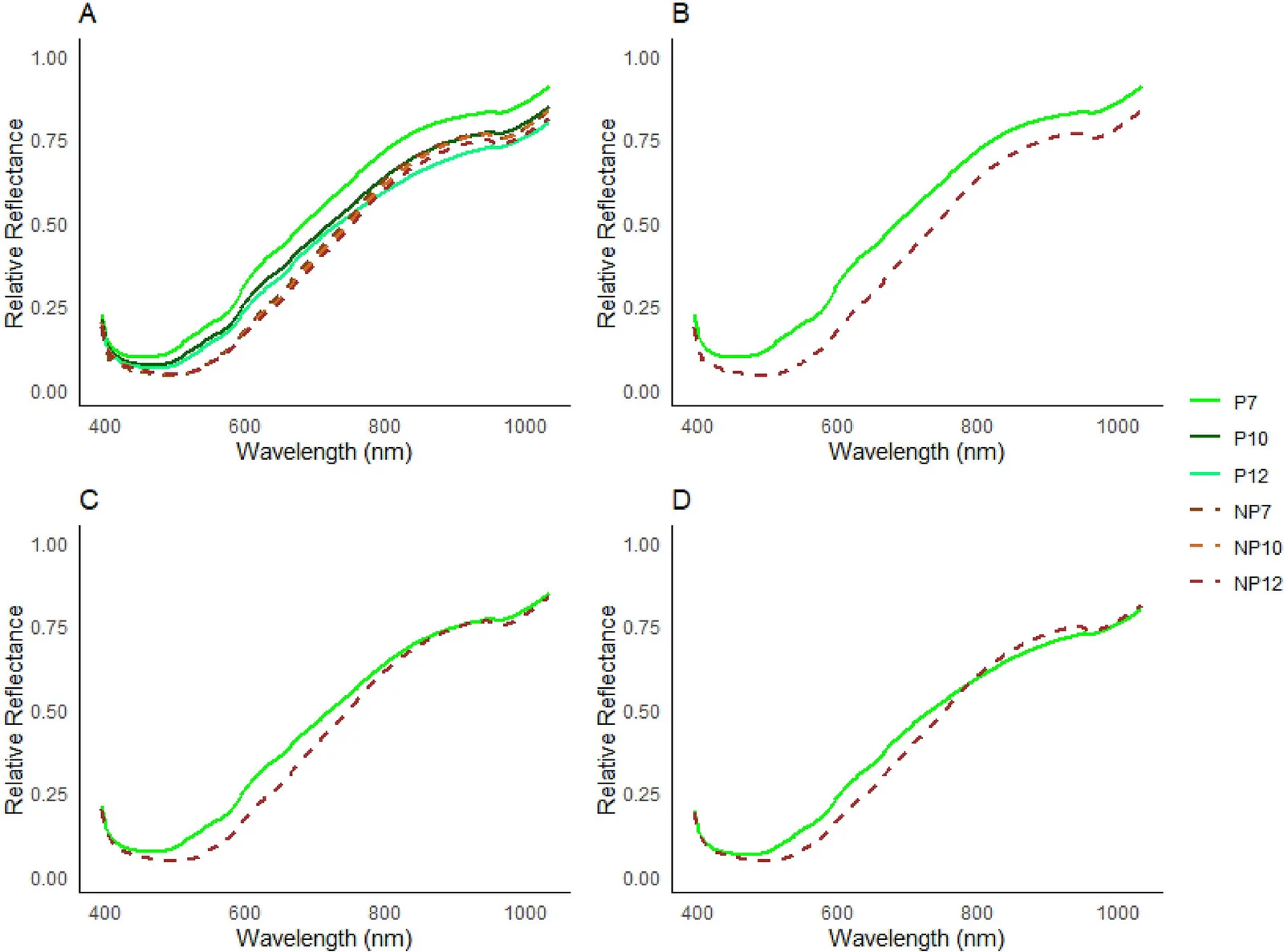
Average spectral profile of parasitized (solid lines) by *Diachasmimorpha longicaudata* and non-parasitized (dashed lines) *Anastrepha fraterculus* pupae evaluated under controlled conditions (26°C and 60% RH). All ages and conditions (**A**). For parasitized pupae (P), 7 days correspond to 135 degree days (DD); 10 days (162 DD), and 12 days (189 DD). For non-parasitized pupae (NP), 7 days (260.1DD), 10 days (290.7 DD), and 12 days (321.3 DD) (**B**, **C**, **D**)

The average spectral profiles (relative reflectance) of *A. fraterculus* pupae parasitized by *D. longicaudata* and non-parasitized at 7 days (135 DD and 260.1 DD, respectively), 10 days (162 DD and 290.7 DD), and 12 days (189 DD and 321.3 DD) are shown in Fig. 3. Across all developmental times (DD) evaluated, the spectra exhibited a consistent pattern, characterized by lower relative reflectance in the visible region (VIS, 400–700 nm) and higher reflectance in the near-infrared region (NIR, > 700 nm) (Fig. 3A).

Parasitized pupae exhibited greater spectral variability across developmental stages than non-parasitized, with this contrast being particularly pronounced at 7 days and near emergence, at 12 days. In 7-day-old pupae (Fig. 3B), reflectance values were consistently higher than those observed at 10 (Fig. 3C) and 12 days (Fig. 3D), a pattern maintained across the entire spectral range. In contrast, non-parasitized pupae displayed lower mean relative reflectance and a more homogeneous spectral profile, with less marked variation among developmental times (DD) (Fig. 3A).

Considering the effect of pupal developmental time (DD), a progressive increase in relative reflectance was observed in both conditions (parasitized and non-parasitized pupae). However, this increase was more pronounced in parasitized pupae individuals, indicating a stronger influence of developmental progression on their spectral response (Fig. 3A).

The performance of the Random Forest model, evaluated using the out-of-bag (OOB) validation strategy, differed among pupal developmental stages (7, 10, and 12 days). Overall, a moderate classification performance was observed, with a progressive improvement in the later developmental stages (Table 1).

**Table 1:** Out-of-bag (OOB) performance metrics of the Random Forest classifier for discriminating parasitized and non-parasitized pupae at different developmental stages (7, 10, and 12 chronological days)

Developmental stage (in days)	OOB Accuracy	OOB Balanced Accuracy	OOB F1-score
7	0.520	0.530	0.509
10	0.547	0.580	0.543
12	0.573	0.590	0.564

**Fig. 4:**
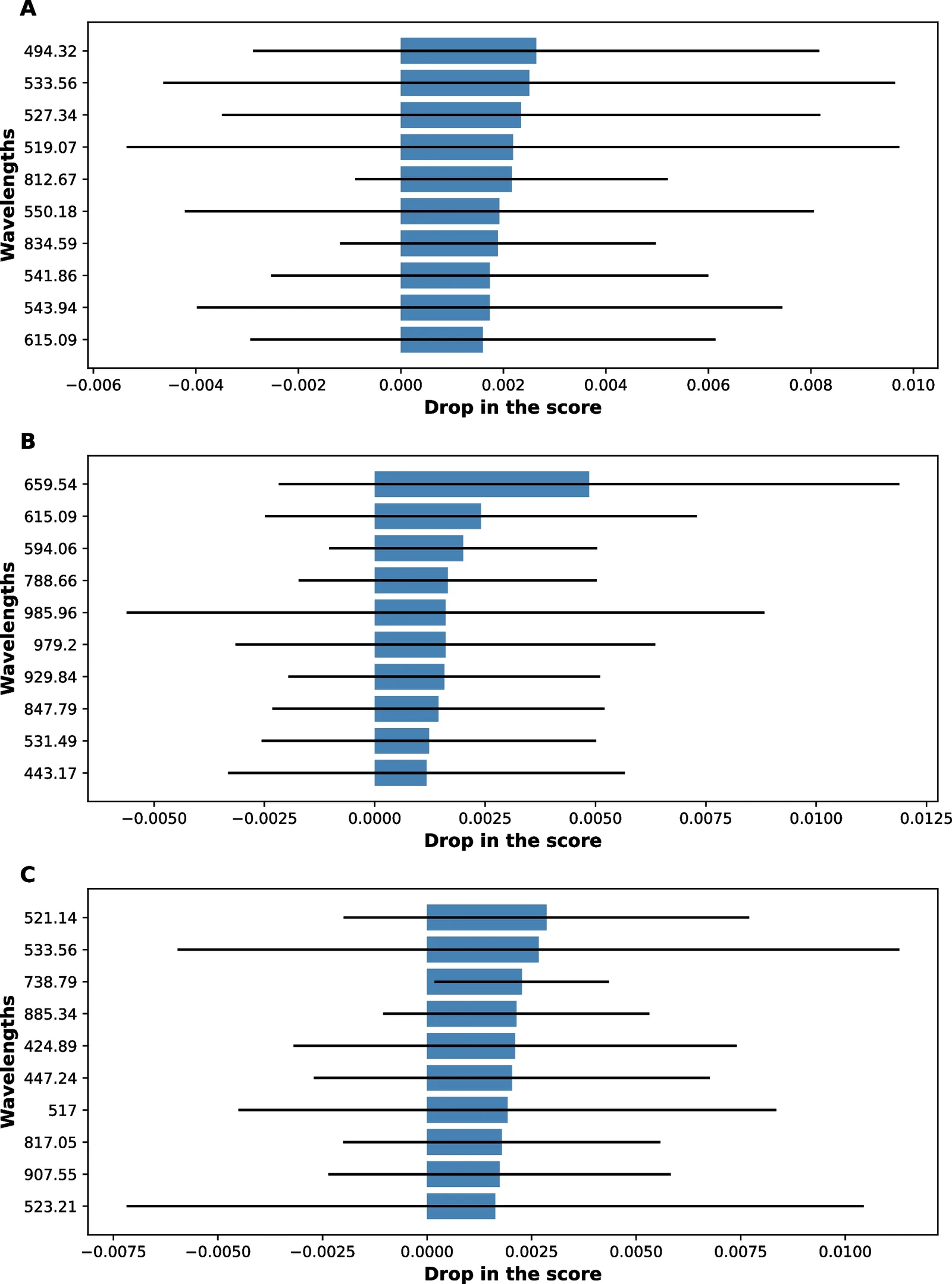
Variable importance of the 10 wavelengths obtained from Random Forest models for pupae at different developmental stages: **A** 7 days (135 DD for parasitized, 260.1 DD for non-parasitized), **B** 10 days (162 DD for parasitized, 290.7 DD for non-parasitized), and **C** 12 days (189 DD for parasitized, 321.3 DD for non-parasitized). Importance was calculated using permutation importance based on out-of-bag (OOB) validation and expressed as the decrease in model performance after permutation of each wavelength. Error bars indicate variability across permutation repetition

The variable importance analysis showed that the spectral relevance varied according to developmental stage, with no single wavelength consistently dominant across all periods evaluated. At all stages, classification relied on the combined contribution of multiple spectral bands distributed across different regions of the spectrum, highlighting the multivariate nature of the discrimination process between parasitized and non-parasitized pupae (Fig. 4).

## Discussion

Principal Component Analysis (PCA) suggested that changes in the spectral patterns between parasitized and non-parasitized pupae became visually apparent from seven days of pupal development onward and persisted across subsequent stages. Similar exploratory patterns in hyperspectral profiles of fruit fly pupae throughout pupal development (expressed in DD) were also reported by Jahnke et al. (2021) and Bender et al. (2025). Although those studies focused primarily on sex differentiation, their results likewise demonstrated that developmental progression strongly influences spectral variability in pupae.

In the present study, spectral profiles were characterized by pronounced absorption (reduced reflectance) in the blue and red regions of the visible spectrum (VIS), followed by increased reflectance in the near-infrared (NIR) region. This pattern is commonly observed in biological materials and reflects variations in both external and internal properties, which may differ according to structure and composition (Zanotta et al. 2019). Specifically, the VIS region (400–550 nm) is largely associated with pigmentation and cuticular characteristics, whereas reflectance in the NIR region (> 700 nm) is more strongly linked to internal biochemical composition, including water, lipid, and protein content (Mastrangelo et al. 2019). Comparable reflectance behavior in the blue region has also been reported for *Drosophila melanogaster* (Meigen, 1830) (Diptera: Drosophilidae), supporting the consistency of these spectral responses across dipteran species (Shibuya et al. 2018).

The results of the present study emphasize the role of parasitism in modulating spectral responses over developmental times. Our findings agree with Nansen and Strand (2018), who observed significant reflectance changes in *Chrysodeixis includens* (Walker, 1858) (Lepidoptera: Noctuidae) larvae parasitized by two parasitoid species between three and seven days after parasitism, while non-parasitized larvae maintained relatively stable reflectance values in the 408–600 nm range. Similarly, the non-parasitized pupae evaluated here exhibited a more uniform and stable spectral profile across wavelengths, likely reflecting physiological homeostasis during normal development. In contrast, parasitized pupae showed greater spectral variability, which may be associated with parasite-induced changes in water balance and in carbohydrate, lipid, and protein metabolism, as previously suggested for parasitized hosts (Nansen and Strand 2018).

Furthermore, parasitized pupae exhibited a pronounced reduction in reflectance at 12 days of development in NIR when compared with non-parasitized pupae of the same age. This progressive decline in reflectance suggests that the physiological impacts of parasitism intensify as pupal development advances. As parasitoids develop within the host, they induce cumulative structural, biochemical, and metabolic alterations that increasingly disrupt normal host physiology, affecting tissue organization, resource allocation, and energy reserves (Dowell et al. 2000). Such parasite-induced modifications can substantially alter the optical properties of the host, resulting in detectable changes in spectral reflectance. Similar patterns of reduced reflectance have been documented in parasitized pupae of several *Drosophila* (Fallén, 1823) (Diptera: Drosophilidae) species relative to non-parasitized individuals (Nansen 2016), as well as in *Chrysomya rufifacies* (Macquart, 1842) (Diptera: Calliphoridae), in which reflectance above 600 nm declined markedly during the final stages preceding adult emergence (Voss et al. 2017). These findings indicate that late developmental stages may represent a period of intensified host–parasitoid interaction, characterized by pronounced physiological stress and altered spectral signatures.

Hyperspectral imaging analyses revealed detectable spectral differences between parasitized and non-parasitized pupae from the early stages of development. Although classification performance was moderate, the identification of distinct spectral patterns from 7 days of development onward (corresponding to 135 DD for parasitized and 260.1 DD for non-parasitized puparia) suggests that hyperspectral approaches can detect subtle physiological and morphological changes associated with parasitism at relatively early developmental stages. Comparable results have not been reported for some alternative imaging techniques for example, X-ray–based studies have shown limitations in detecting parasitism in young *A. fraterculus* pupae between three and seven days of development (Marinho et al. 2023), highlighting the sensitivity of optical spectral information to surface- and composition-related changes.

The variable importance analysis further demonstrated that spectral relevance varied according to developmental stage, with wavelengths located in the visible (VIS) region frequently ranked among the most informative features. However, wavelengths in the near-infrared (NIR) region also contributed to classification, particularly at intermediate and later stages. Importantly, no single wavelength or spectral region was consistently dominant across all evaluated stages. Instead, discrimination relied on the combined contribution of multiple spectral bands, reflecting the inherently multivariate nature of hyperspectral data. Similar spectral intervals, such as 408–600 nm, have also been reported as relevant for parasitism detection in other insect–parasitoid systems (Nansen and Strand 2018), reinforcing the consistency of these spectral patterns across different biological contexts.

The stage-dependent variation in the most informative wavelengths underscores the importance of considering pupal age in spectroscopic analyses. Identifying relevant spectral ranges and appropriate temporal windows is essential not only for improving discrimination between parasitized and non-parasitized pupae but also for guiding the development of more applicable detection systems. From a practical perspective, this information may support the design of simplified spectroscopic devices operating with a reduced number of spectral bands, potentially reducing acquisition time and computational demand.

Although such systems could, in principle, be integrated into biofactory environments for automated pupal sorting, additional studies are required to validate model robustness under operational conditions. Challenges related to biological variability, spectral signal overlap, sample orientation, and real-time processing must be addressed before large-scale implementation. Nonetheless, the present results highlight the potential of combining hyperspectral imaging with machine-learning approaches as a promising nondestructive tool for assessing parasitism in insect pupae under controlled conditions.

## Data Availability

The datasets supporting the results of this study are available from the corresponding author upon reasonable request.
